# T-cell Prolymphocytic Leukemia Presenting as Red Eye

**DOI:** 10.4103/0974-9233.75896

**Published:** 2011

**Authors:** Fahad Alwadani

**Affiliations:** Department of Ophthalmology, King Faisal University, King Fahad Hospital of the University, PO Box 2208, Al-Khobar 31952, Saudi Arabia

**Keywords:** Conjunctival Injection, Prolymphocytic Leukemia, Red Eye

## Abstract

T-cell prolymphocytic leukemia (T-PLL) is a rare, highly aggressive, mature T-cell neoplasm. Ocular involvement in T-PLL is very rarely described in the literature. There are only two reports in the literature documenting conjunctival involvement in cases with T-PLL. Conjunctival involvement may be the presenting sign of the disease or rarely signifies the relapse of the disease. We present a case of a 36-year-old Saudi male patient in whom bilateral red eyes were the presenting sign of T-PLL.

## INTRODUCTION

T-cell prolymphocytic leukemia (T-PLL) is a rare, mature T-cell neoplasm that is rapidly progressive and more aggressive than B-cell prolymphocytic leukemia (B-PLL).^1^ T-PLL has a median survival of 7.5 months.^2^ T-PLL affects adults and occurs more frequently in men. The main features of the disease at presentation are lymphadenopathy, hepatosplenomegaly, skin lesions and marked leukocytosis.^1^ T-PLL expresses mature T-cell markers (TdT−, CD1a−, CD2+, CD3+, CD5+, strongly CD7+ and CD52+), usually of a T-helper phenotype (CD4+, CD8−). Infrequently, T-PLL may immunophenotype as CD4−/CD8+, and in 25% of cases, the cells may co-express both CD4 and CD8, a feature almost unique to this entity.^1^,^3^

Although studies have reported an incidence of 50–90% ocular and orbital involvement in other types of leukemia,^4^ ocular involvement with T-PLL is rare.^3^ We report the clinical findings of possible conjunctival involvement in a case with T-PLL.

## CASE REPORT

**Figure 1:**
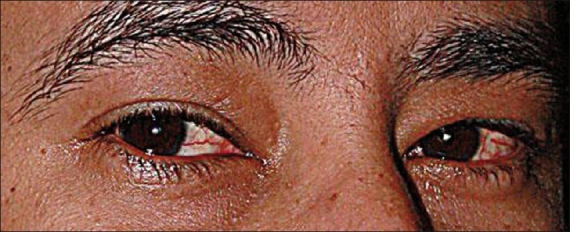
Bilateral red eyes

A 36-year-old male Saudi patient presented to a private ophthalmic clinic, complaining of red eyes bilaterally [Figure 1]. He had no past history of systemic or ocular diseases. On examination, the visual acuity was 20/25 bilaterally. Extraocular motility was normal and there was no proptosis. Slit-lamp examination showed diffuse conjunctival injection and mild chemosis. There were no elevated lesions or other inflammatory signs. Anterior and posterior segments were unremarkable. Regional lymphadenopathy was not detected. The patient was diagnosed with irritative conjunctivitis. Lubricants and soothing agents were prescribed to the patient with a follow-up scheduled for 2 weeks after presentation.

One month later, he presented to the emergency room with abdominal cramps, skin eruptions, malaise, and fatigue, but no fever, shortness of breath, chest pain or bleeding. Examination showed pallor and generalized external lymphadenopathy. His lymph nodes were discrete, 0.5–1 cm in diameter, firm and not tender. He had leg edema and papular skin rash around the neck. His chest was clear with reduced air entry over the right lower zone. Cardiovascular examinations revealed normal heart sounds. The spleen was palpable, 4 cm below the costal margin, the liver was impalpable and there was no clinically detected ascites.

**Figure 2:**
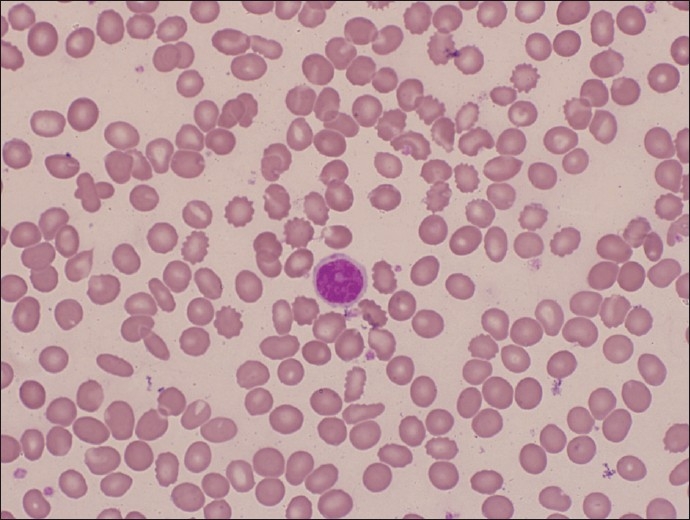
Circulating prolymphocyte with thrombocytopenia

Ocular examination showed persistence of conjunctival injection, 20/25 visual acuity with no other abnormal ocular findings. His blood count was as follows: WBC count was 403 × 10^9^/L; hemoglobin 141 g/L; and platelets 51 × 10^9^/L. His blood film showed normocytic normochromic red cells, moderate thrombocytopenia, leukocytosis with predominance of lymphoid cells that resembled prolymphocytes with medium size and prominent centrally located nucleoli [Figure 2]. His renal, hepatic and coagulation profiles were all normal.

Bone marrow examination showed a hypercellular marrow, which was heavily infiltrated (95%) with lymphoid cells described earlier in the blood film. Trilineage hematopoiesis was markedly suppressed. There was markedly increased reticulin stain. The working diagnosis favored T-PLL. Computed tomography (CT) scan of the chest, abdomen and pelvis showed extensive internal lymphadenopathy in the mediastinal, hilar retroperitoneal, mesenteric and pelvic areas with moderate degree of ascites and large right-sided pleural effusion with right lower lobe collapse in addition to splenomegaly. Magnetic resonance imaging (MRI) of the orbit was normal.

After confirming the diagnosis of T-PLL, a peripherally inserted central catheter line was inserted and the patient commenced the FC regimen composed of fludrabine 30 mg/m^2^ IV for 3 days and cyclophosphamide 300 mg/m^2^ IV for 3 days. He was also given IV fluids for hydration; allopurinol, ranitidine and bactrim, acyclovir and fluconazole for prophylaxis against infection.

Different combinations of topical lubricants and anti-inflammatory topical eyedrops were prescribed to relieve conjunctival congestion and chemosis, without success. However, dramatic response with complete resolution of the conjunctival congestion and chemosis was detected within 4 weeks after beginning the FC regimen. A conjunctival biopsy was not performed.

## DISCUSSION

Ocular infiltration with leukemia most commonly affects the retina and choroid.^5^ However, conjunctival leukemic infiltration has been previously frequently described.^6^–^8^ Conjunctival leukemic infiltration was first published by Leber in 1878.^4^,^9^ Conjunctival leukemic infiltration occurs most commonly with acute leukemia. Diffuse infiltration is the most common presentation, but focal lesions and limbal involvement have also been documented.^4^

T-PLL is a rare, chronic, mature T-cell neoplasm that represents less than 20% of PLL (the remaining 80% is B-PLL).^1^ The clinical features, morphological variations and cytogenetic characteristics were reported by Matutes *et al*. in 1991.^2^ Ocular involvement in PLL is very rarely described in the literature.^4^,^10^–^13^ One case presented with subretinal hypopyon-like appearence,^11^ another case presented with bilateral periorbital swelling^12^ and another case presented with panuveitis.^13^ Conjunctival involvement with T-PLL has been previously described only twice.^4^,^10^ Naseem *et al*.^10^ describe a case of T-PLL that presented with bilateral conjunctival hemorrhage and no other ocular abnormalities. Conjunctival biopsy was not performed and the patient was lost to follow-up.

In 2004, Lee *et al*.^4^ described the only histologically proven case of conjunctival involvement with T-PLL. Their^4^ patient was a 53-year-old African-American who had chronic red eye approximately 6 months after relapse of T-PLL that was previously diagnosed and successfully treated 4 years prior. The red eye was originally diagnosed as ocular hypertension and continued to progress over a period of weeks.^4^ Examination of the patient revealed bilateral symmetrical proptosis, and bilateral 360°, perilimbal, slightly elevated and indurated conjunctival lesion that extended 3–5 mm posterior to the limbus. Visual acuity was 20/25 and no other ocular abnormalities could be detected with exception of bilateral mild tortuosity of retinal vessels.^4^ MRI showed a 1-cm retro-orbital mass adjacent to the globe in the right eye.^4^ Conjunctival biopsy showed atypical lymphocytes.^4^ Immunophenotyping of the cells was consistent with the diagnosis of T-PLL. Clinically, there was complete resolution of the conjunctival infiltration over an 8-week period after initiating retreatment.^4^

Our case differed from Lee *et al*.’s^4^ case. In our case, the patient developed diffuse red eyes. No elevated lesions were present in the conjunctiva during the period of diagnosis and treatment of T-PLL. This is may be due to the early treatment that controlled the disease before progression of elevated lesions into the conjunctiva. Lee *et al*.^4^ reported the development of perilimbal elevated lesions many weeks after the onset of diffuse red eye. Autopsy studies of leukemia patients indicate that many haror conjunctival infiltration that was not diagnosed premortem,^3^ indicating that conjunctival involvement in leukemia may be subclinical or may mimic mild conjunctivitis. In fact, there is no record of reduction of vision in all reported cases with of T-PLL with conjunctival involvement.^4^

In our case, conjunctival injection developed 1 month prior to any signs or symptoms that suggest leukemic infiltration of the conjunctiva. Review of the literature showed that conjunctival involvement may be a presenting sign of leukemia or signifies relapse of the disease.^2^–^4^,^6^–^8^

There is no histopathological proof of conjunctival infiltration with leukemia in our case. This was due to the rapid diagnosis of the disease and the rapid response to treatment. However, the resistance of the conjunctival injection and the rapid and complete resolution after starting chemotherapy coupled with the fact that many cases with conjunctival leukemia can mimic simple conjunctivitis indicates that conjunctival injection in our case was due to leukemic infiltration.

In conclusion, we report to the best of our knowledge the first case of conjunctival involvement with T-cell prolymphocytic leukemia to be recorded in the Middle East. It is a rare cause of conjunctival injection that was treated effectively with chemotherapy.
